# Direct and rapid mass spectral fingerprinting of maternal urine for the detection of Down syndrome pregnancy

**DOI:** 10.1186/s12014-015-9082-9

**Published:** 2015-03-24

**Authors:** Ray K Iles, Maryam E Shahpari, Howard Cuckle, Stephen A Butler

**Affiliations:** MAP Diagnostics Ltd, The BioPark, Broadwater Road, Welwyn Garden City, Hertfordshire, AL7 3AX UK; Middlesex University, Hendon, London, UK; Columbia University Medical Center, New York, USA

**Keywords:** NIPT, Down syndrome, MALDI ToF MS, Urinalysis

## Abstract

**Background:**

The established methods of antenatal screening for Down syndrome are based on immunoassay for a panel of maternal serum biomarkers together with ultrasound measures. Recently, genetic analysis of maternal plasma cell free (cf) DNA has begun to be used but has a number of limitations including excessive turn-around time and cost. We aimed to develop an alternative method based on urinalysis that is simple, affordable and accurate.

**Method:**

101 maternal urine samples sampled at 12–17 weeks gestation were taken from an archival collection of 2567 spot urines collected from women attending a prenatal screening clinic. 18 pregnancies in this set subsequently proved to be Down pregnancies. Samples were either neat urine or diluted between 10 to 1000 fold in dH_2_O and subjected to matrix assisted laser desorption ionization (MALDI), time of flight (ToF) mass spectrometry (MS). Data profiles were examined in the region 6,000 to 14,000 m/z. Spectral data was normalised and quantitative characteristics of the profile were compared between Down and controls.

**Results:**

In Down cases there were additional spectral profile peaks at 11,000-12,000 m/z and a corresponding reduction in intensity at 6,000-8,000 m/z. The ratio of the normalised values at these two ranges completely separated the 8 Down syndrome from the 39 controls at 12–14 weeks. Discrimination was poorer at 15–17 weeks where 3 of the 10 Down syndrome cases had values within the normal range.

**Conclusions:**

Direct MALDI ToF mass spectral profiling of maternal urinary has the potential for an affordable, simple, accurate and rapid alternative to current Down syndrome screening protocols.

## Background

Currently, prenatal screening for fetal Down syndrome is based on the quantitative measurement of up to four maternal serum biochemical markers (the principle component being hCG) at 15–18 weeks of gestation or two serum markers and ultrasound nuchal translucency at 11–13 weeks of gestation. Increasingly, the latter approach is used because of a higher, 80-90%, detection rate for a 5% false positive rate together with the option of first trimester diagnosis and termination of affected pregnancies [1].

Recently cell free (cf) DNA, isolated from maternal blood, has been developed as a new screening test for Down syndrome with a greater than 99% detection rate and less than 0.5% false positive rate [2]. Since this test can substantially reduce the need for invasive prenatal diagnosis, it is often described as a non-invasive prenatal test (NIPT). Commercially available cfDNA tests will also detect other common types of aneuploidy. They use a variety of analytic methods including shotgun massively parallel sequencing, targeted sequencing and single nucleotide polymorphisms together with complex algorithms [3,4]. Technical limitations such as a turnaround time exceeding a week, a high test failure rate, and high cost [5] preclude immediate implementation in a public health setting, although costs are reducing all the time and methods are improving. Other methods of quantifying cfDNA have been reported including methylated DNA-based approaches [6] and digital PCR [7].

In the mid 1990’s maternal urine analysis and in particular the quantitative immunoassay of hCGβcf was proposed as an alternative to Down syndrome screening based on maternal serum markers. Initial studies were encouraging as maternal urine levels of women carrying a fetal aneuploidy were on average 5–6 times higher than pregnancies with non-aneuploid fetuses [8-14]. Thus, urinary hCGβcf was considered to be potentially superior to serum hCG which was on average only increased about two-fold. However, not only did hCGβcf levels need to be corrected for gestational age, but adjustment had to be made for the dilutional effects of water intake and urination [15]. This meant that simple quantitated levels of hCGβcf per unit volume were so variable that performance as a single screening marker was not substantially greater, in terms of discriminatory power, than existing serum based analysis [16-18].

Although this variability can be corrected for by methods such as expressing levels as a ratio of another urinary marker, such as creatinine, this did *not* prove satisfactory. Indeed, since creatinine is influenced by body mass and increased metabolism, it was never an ideal correction factor for pregnant women. Although other corrections are possible they were neither robust nor sufficiently feasible to be practical [15].

Matrix Assisted Laser Desorption Ionisation Time of Flight Mass Spectrometry (MALDI-ToF MS) can allow the examination of genetic and metabolic diseases at the level of protein expression and where posttranslational modifications of specific proteins cause variations in molecular mass to occur [19]. Therefore, proteomics and genomics can be linked, helping in the diagnosis and therapeutic monitoring of reproductive clinical disorders [20].

Recently, we have used MALDI-ToF MS to identify variation in the glycosylation that occurs in human chorionic gonadotrophin beta core fragment (hCGβcf), following reports of an increase in hCG hyper glycosylation in Down syndrome pregnancies [21-23]. We adopted a non-direct approach to analyse molecules in the high-resolution range of 3,000 -5,000 m/z [22,24]. Differences in the mass spectral profile in this mass region were indicative of disorders in pregnancy [25].

Although we proposed that such variation might form the basis of a clinical diagnostic test for abnormal pregnancies, the purification of proteins from large urine collections is not feasible in a clinical diagnostic situation. However, the advances of MALDI-ToF MS instrumentation (with a flight tube of 1 meter or more) can now resolve abundant urinary molecules in the inferred mass region of 6,000 to 15,000 m/z without the need of any purification.

We examined the mass spectral profiles of maternal urinary metabolites in the 6,000 to 15,000 m/z region in single, dH_2_0 diluted, urine samples from pregnant women with Down syndrome and those whose pregnancy was known not to be affected by fetal aneuploidy (controls). The ability to use this post genomic mass spectrometry approach as a potential rapid, robust and affordable Down syndrome screening test was explored.

## Results and discussion

Urine analysis has seen a resurgence of interest with the advent of proteomics and metabolomics coupled to technological developments in mass spectrometry techniques [26,27]. Conceptually the focus is moving from a quantitative measure of any given biomolecule(s) but to compositional profiles [28,29] and the more subtle post translational modification of proteins – the proteoform - that are markers of a disease process [25].

Approaching the analysis of maternal urine with a view that a qualitative structural change to a molecule, rather than a quantitative change is the marker of disease, circumvents the problems associated with urine analysis for Down syndrome highlighted twenty years ago.

**Figure 1:**
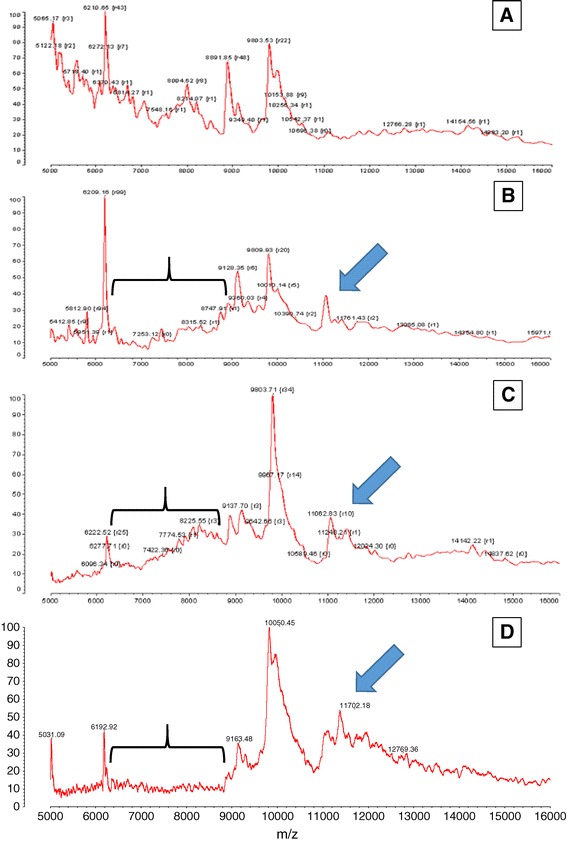
**Example MALDI Tof mass spectral profiles of maternal urine between ~ 5,000 and 16,000 m/z. A**: non-aneuploid pregnancy at 13,4 weeks gestation; **B**, **C & D**: Trisomy 21/Down syndrome, aneuploidy samples at 13,3 Weeks, 12,4 weeks and 16,6 weeks gestation. Large blue arrows indicate regions of additional peaks at 11,000 – 12,000 m/z and the orthogonal stretched bracket indicate regions of ~ 6,500 – 8,750 m/z that are less abundant in the Down syndrome samples.

Visual examination of non-normalized MALDI-ToF MS spectral profiles of maternal urine between ~5,000 and 16,000 m/z revealed substantial differences between Down syndrome and controls. Figure 1 illustrates this with the profiles of three Down syndrome cases and one control. In Down syndrome there are additional peaks at 11,000–12,000 m/z and apparent loss of peaks at ~6,500–8,750 m/z. This effect was apparently greater at 12–14 than 15–17 weeks.

We have previously demonstrated that the post-translational glycosylation of hCG produces a series of glycoforms that are altered in placental disease and when expressed by cancer [30-34]. Furthermore, these glycoform differences are reflected in hCG’s major urinary metabolite hCGβcf, which can be detected by MALDI-ToF mass spectrometry [22,25]. However, it was presumed that purification and often concentration of the urine sample would be necessary [35,36]. Contrary to this, since hCGβcf is the most abundant glycoprotein in maternal urine we found that neat and preferably dilution of maternal urine resulted in reproducible mass spectral profiles in the mass regions expected for hCGβcf.

**Figure 2:**
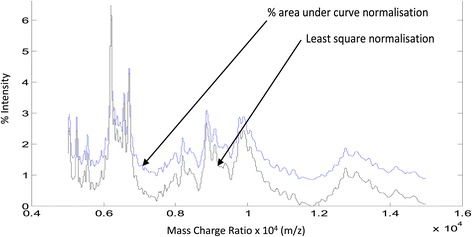
**Effect of normalisation of spectral intensity of an example urine sample at 5,000 to 15,000 m/z: Plot of blue line - % area under the curve and black line - least square normalisation, of percentage intensity per 100 m/z bin units.**

In order to numerate the visual changes and compare them statistically, the spectral data was captured between 6,000 m/z and 14,000 m/z. We choose to quantitate the MALDI-ToF MS in bins of 100 m/z which allows for huge variation in calibration and machine accuracy (although MALDI is often quoted as <0.2% CV in mass/charge measurement) and normalised as described. Both methodologies kept the basic profile; however, as well as reducing the base-line, normalisation by the least square method emphasised the peaks (see Figure 2).

**Figure 3:**
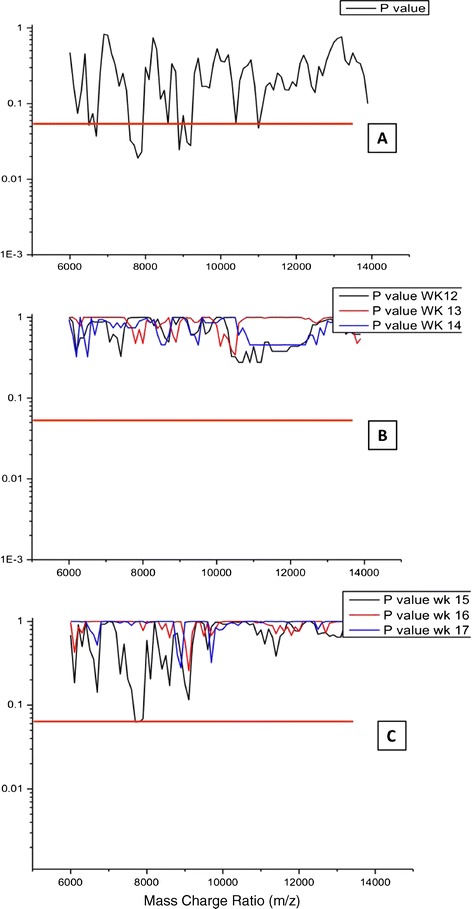
**Examination of the variability in m/z profiles from 84 non-aneuploid maternal urine samples as gestation progresses from 12 to 17 weeks of pregnancy.** Plot of statistical significance comparisons (p-values) of each mass bin (every 100 m/z from 6000 to 14000 m/z) of normalised data from maternal urinary MALDI – ToF mass spectral profiles. The red lines indicate a statistical significance cut off at p = 0.05. **Panel A** – kruskall Wallis test comparison of all samples grouped from 12 – 18 weeks gestation, illustrating multiple m/z regions where there is a difference between the profiles as gestation progresses; **Panel B** – step wise comparisons between 12, 13 and 14 week gestation samples, illustrating that at these gestations there are no differences in the profiles of the non-aneuploid samples; **Panel C** – step wise comparisons between 15, 16 and 17 week gestation samples, illustrating differences at specific m/z regions but none reach statistical significance at p < 0.05.

The samples collected crossed a major maternal and fetal-placental physiology boundary between the first and 2nd trimester of pregnancy [37]. In order to see what affect this had on the mass spectral profile, normalised spectral data was compared between samples at 12 and 17 weeks gestation in the non-aneuploidy pregnancy. The median and associated variance of normalised spectral area values were calculated at each of 100 m/z bins between 6,000 and 14,000 m/z. As illustrated in Figure 3, by plotting of p – values against m/z bin, Kruskall Wallis statistical analysis revealed significant differences (p < 0.001) between the normalised sample spectra as gestation age progressed. Indeed this indicated that particular regions e.g. 7,000 – 8,000 and 8,900- 9,400 m/z were very variable (Figure 3A). However, if the transition from first to 2nd trimester was delineated by separating samples of 14 weeks gestation or less from those of 15 weeks or greater; Kruskall wallis analysis showed that there was no significant differences in profile mass between the normal non-aneuploid samples at any m/z bins if delineated by a 14 week cut off: Examining pair-wise comparisons, the normalised profiles at 12–14 weeks gestation were remarkably consistent and clearly not significantly different from each other (see Figure 3B). Non-aneuploid pregnancy samples at 15, 16 and 17 weeks gestation were not significantly different from each other however they were not as similar as those of earlier gestation (see Figure 3C). Indeed, in particular spectra of samples at 15 week gestation approached, but did not reach statistical significant differences from 16 and 17 week gestational samples at 7,000 – 8,000 and 8,900 - 9,400 m/z.

Given the changes in profile with gestational age the sample sets were divided into two groups; samples of 12 to 14 weeks gestation and samples of 15–17 weeks gestation (see Table 1) for comparative analysis of profile changes that were associated with a Down pregnancy.

**Table 1:** **Maternal urine samples according to gestation (nearest week) in Down syndrome and controls**

**Gestational (weeks)**	**Down’s syndrome**	**Controls**
12	0	3
13	8	26
14	0	10
**12-14**	**8**	**39**
15	1	15
16	8	19
17	1	10
**15-17**	**10**	**44**

Numbers in bold indicate subtotals at 12-14 and 15-17 weeks gestation.

**Figure 4:**
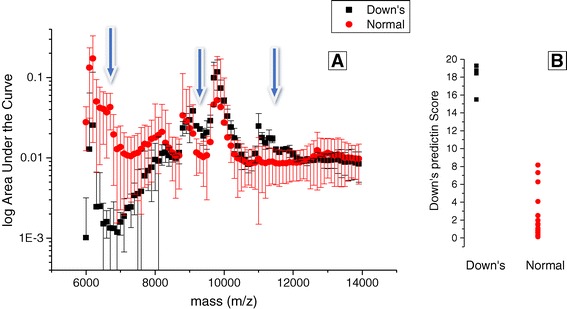
**Comparison based on least squared normalization technique for Down and non-aneuploid pregnancies at 12–14 weeks gestation: A - shows overlaid average mass spectra with bin median as solid shapes and 5 and 95th centiles as whiskers for Down (black squares) and non-aneuploid (red circles) pregnancies at 6,000 to 14,000 m/z.** The arrows indicate areas of maximal separation. **B** - a dot plot of the application of a simple predictive algorithm score called a Down Predictive Score = y(m/z 11400) + y(m/z 9200) / y(m/z 6700) based on the spectral differences of the eight Down syndrome and 39 non-aneuploid samples, indicating potential 100% detection and 0% false positive rate.

Normalised spectral data was compared between 8 trisomy 21/Downs samples and 39 non-aneuploidy pregnancy urine samples at 12–14 weeks of gestation (see Figure 4). The % area under the curve and least squared normalisation yielded near identical result with marginally less variation around the medians for the least squared normalised data. By plotting the median values, 5th and 95th centiles as whiskers it was evident that Down syndrome spectra varied significantly from that of non-Down pregnancies (Kruskal wallis test p < 0.001). Spectral values were dramatically lower than those of non-aneuploidy samples at 6,000 to 8,000 mz and higher at ~9,400mz and 11,000 – 11,500 m/z (Figure 4A). Creating a comparable quantitative numerate value for the y measure meant that computational comparative scoring can be created. A simple algorithm was created that distinguished Trisomy 21/ Down pregnancy from non-aneuploidy (score = y(m/z 11,400) + y(m/z 9,200) / y(m/z 6,700) with a 100% sensitivity and specificity. Indeed, the margin of separation was so large that seven-integer number cut off scores could be applied with the same accuracy (see Figure 4B).

**Figure 5:**
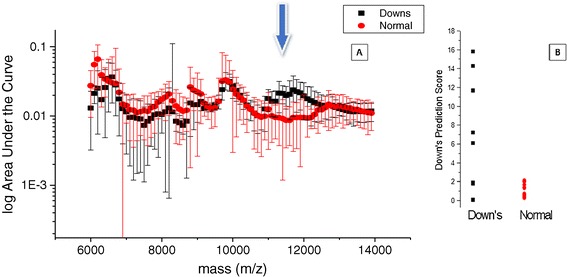
**Comparison (based on least squared normalization) of Down and non-aneuploid pregnancies at 15–17 weeks gestation: A - shows overlaid average mass spectra with bin median as solid shapes and 5 and 95th centiles as whiskers for Down (black squares) and non-aneuploid (red circles) pregnancies at 6000 to 14,000 m/z.** The arrow indicates areas of maximal separation. **B** - a dot plot of the application of a prediction algorithm score called a Down Predictive Score = Σ y(mz11300 to mz11900) based on the spectral differences of the 10 Down syndrome and 44 non-aneuploid samples indicating potential 70% detection for a 0% false positive rate.

Normalised spectral data was also compared between 10 trisomy 21 Down samples and 44 non-aneuploidy pregnancy urine samples at 15–17 weeks of gestation (see Figure 5). The % area under the curve and least squared normalisation yielded near identical results. By plotting the median values, 5th and 95th centiles as whiskers it was evident that Down syndrome spectral values were still statistically different (p < 0.001) to those of non-aneuploidy samples at 6,000 to 8,000 mz and the profiles, whilst consistently lower, were merging in this region (Figure 5A). However, values remained higher in the Down samples at m/z of 11,000 to 12,000. Nevertheless, the comparable quantitative numerate values for the y measure still meant that computational comparative scoring was still possible. In a simple algorithm was created that distinguished Trisomy 21/ Down pregnancy from non-aneuploidy (score = Σy(m/z 11,300) to y(mz11,900)) with a 70% sensitivity and 100 specificity at 15–17 weeks gestation.

The experiments here demonstrate that no processing, as we had previously employed [22,25], was necessary to yield a sufficiently resolved spectra. This is due predominately to using longer ToF flight tubes and advances in analysis technology which yields high resolution in the 10,000 to 20,000 m/z range. Furthermore, rather than rely on an operator subjective evaluation of a mass spectra, MALDI-ToF mass spectra can be rendered semi-quantitative such that automated quantification of the qualitative change in the proteform pattern can be analysed. Also the spectral profiles in unaffected pregnancies do not appear to differ markedly according to gestational age in the first trimester of pregnancy, such that exacting ultrasound dating would probably not be a prerequisite of testing. While we acknowledge that the samples are quite old, very little variability in this mass region has been shown in stability studies. This is probably due to the nature of the molecules being examined, and while hCG in urine does degrade into subunits [38] terminal metabolic degradation products do not further degrade when stored at −20°C, even for longer periods of time. Even so, this may represent a significant limitation to this study.

In summary, urinary mass spectral profiles differed in the pattern of protein and glyco-peptide molecules detected in the urine of women carrying a fetus with Down syndrome. Focussing on simple MALDI-ToF analysis of neat or diluted urine we propose a mass spectral pattern analysis test that has the potential to achieve a very high Down syndrome detection rate (>99%) with a low false positive rate (<1%) in the first trimester of pregnancy. In later second trimester performance was not as good with 3 of the Down samples being missed. In receiver operator plots a compromise of 90% detection for 6% false positive can be achieved (data not shown); but larger numbers of samples and more sophisticated scoring algorithms would optimise this evaluation approach for screening for Down syndrome.

We have used archival stored urine and a relatively small series of Down syndrome cases so more studies on fresh and diverse samples are needed. On the other hand we have used a simple way of interpreting the spectral profile as a ratio of areas under the curve and it is likely that a more sophisticated mathematical model would yield better result.

Such a proposed analytical approach has the distinct advantages over current Down syndrome screening protocols in terms of turn-around time and ease of sample collection. Results can be generated in minutes rather than days and the patient can collect the sample at home. It also has a major advantage over the new cfDNA screening test in terms of logistics and cost; In order to recover sufficient cfDNA, maternal blood sampling must be after 10 weeks gestation and so requires ultrasound dating. Even at later gestations, there is a test failure rate largely due to insufficient fetal cfDNA in samples. Turn-around time exceeds a week and with repeat testing, in some cases much longer. The commercial cost of cfDNA testing varies from $900 to more than $2500 in the USA and about half this in other countries. Whilst cfDNA costs are reducing, the urinary test we propose here would likely be a fraction of this cost. Thus, the potential is for a MALDI–ToF mass spectra based test of maternal urine, to be simple, robust and affordable.

## Methods

### Pregnancy urine samples

An archive of 2537 maternal urine sample was collected in 1993–2000 from women attending a large teaching hospital for Down syndrome screening. Participating women were recruited to a research program designed to investigate potential urinary markers of aneuploidy [9-12]. A single early morning urine sample was collected from each woman, aliquoted and stored at −80°C. Urinary marker results were not used clinically. Information on aneuploidy status was subsequently obtained from the records of the local cytogentics laboratory. Permission to access anonymized samples from the archived set was granted by the ethics committee of Middlesex University (Natural Sciences Ethical approval number 347).

For the current MALDI-ToF MS analysis 101 urine samples at 12–17 weeks gestation were identified: 18 were from Down syndrome pregnancies and 83 were from unaffected controls. Table 1 shows the distribution of gestations. The urine samples were removed from the freezer and allowed to thaw on ice. Samples were centrifuged for 3 minutes at 1500 rpm in order to remove any particulate debris and 100 μl aliquots prepared and re-frozen at −20°C until used.

### MALDI-ToF mass spectrometry

Steel MALDI plates (384 wells) were prepared by pipetting 0.5 μl of sinapinic acid matrix solution (20 mg/ml dissolved in 50/50 v/v acetonitrile (ACN)/ddH_2_O and 0.1% trifluoacetic acid (TFA)) and allowed to dry. 0.5 μl of urine, and urine diluted 1/10 to 1/1000 in distilled deionised water, was spotted on the dry matrix, followed by the addition of a further 0.5 μl of sinapinic acid matrix solution. This was allowed to dry at room temperature for 1 hour before MALDI TOF MS analysis.

The mass spectrometric analysis was carried out using a Shimadzu Axima CFRplus MALDI mass spectrometer: the pulse nitrogen laser (λ_max_ = 337 nm), was fired at 90% power. The ions were accelerated by a 20 kV electrical field down a 1.2 m linear tube and detected by a micro-channel plate detector at a sampling rate of 500 MHz. Spectra were generated by summing 20–30 laser shots. A positive linear mode was used in order to acquire the spectra.

Mass calibration was assigned using horse heart cytochrome C at a concentration of 10 pmol/μl as an external calibrant. The two points calibration generated was at [M + H]^+^ = 12 361 Da and [M + 2H]^2+^ = 6,181 Da for the spectral analysis of whole hCGβcf. Data was exported from the spectral fingerprint between 6,000-14,000 m/z.

### Spectral data normalisation

In order to compare the spectra, normalisation was necessary as Y axis values are relative to maximum signal detected and not a measured scale. Thus, the algorithms developed (below) and used in this study to normalise the raw data allowed us to compare relative intensity values in the spectra. Thus, data generated, peak mass and intensity could all be compared and utilised in a meaningful and automated computational manner. In order to do this, the total area under the curve (which is the integration of each sample within the mass range of 6,000-14,000 m/z) was calculated and the same region was divided into 80 individual bins of 100 m/z unit.

The area under the curve of each bin was calculated and expressed as a percentage of total spectral area under the curve of 6000–14000 m/z based on the formula: $\mathrm{Y}\ \%\ \mathrm{difference}=\frac{\left(y1-y\kern0.5em ref\right)}{y\kern0.5em ref} \times 100\%$, where y1 is the area value at a given 100 m/z point (bin) and y ref is the total area under the curve.

In an alternative normalization approach, the spectral area relative intensity (Y Axis value) is calculated by the “least square of difference “. In this method, the minimum bin Y value of the spectra (y refmin) was subtracted from the Y value at every 100 Da point (bin) and the difference was squared. The formula used to calculate square of difference = (*y*1 − *y refmin*) ^ 2.

### Statistical analysis

The median and 5-95th centile range deviation of normalized intensity values was calculated for Down syndrome and control samples at each bin and for groups of bins within a specified range. Statistical significance of the difference between these groups of samples was evaluated using Kruskal-Wallis (Conover-Inman) and when appropriate, Mann–Whitney U tests.

Since the urine maternal and fetal physiology and anatomy is continually changing with gestation [29,37], any affect on spectral profile was assessed by comparing the normalised values between 12–14 week and 15–17 week samples at each bin.

Data were recorded in Excel 2007 and statistical analyses performed with Stats-Direct™ software. Tests with p ≤ 0.05 were classified as statistically significant.
